# Labor productivity and employment gaps in Sub-Saharan Africa

**DOI:** 10.1016/j.foodpol.2016.09.013

**Published:** 2017-02

**Authors:** Ellen B. McCullough

**Affiliations:** Department of Agricultural & Applied Economics, University of Georgia, United States

**Keywords:** Structural transformation, Sub-Saharan Africa, Agricultural labor productivity, Sector labor shares, Productivity gaps

## Abstract

Drawing on a new set of nationally representative, internationally comparable household surveys, this paper provides an overview of key features of structural transformation – labor allocation and labor productivity – in four African economies. New, micro-based measures of sector labor allocation and cross-sector productivity differentials describe the incentives households face when allocating their labor. These measures are similar to national accounts-based measures that are typically used to characterize structural change. However, because agricultural workers supply far fewer hours of labor per year than do workers in other sectors in all of the countries analyzed, productivity gaps shrink by half, on average, when expressed on a per-hour basis. Underlying the productivity gaps that are prominently reflected in national accounts data are large employment gaps, which call into question the productivity gains that laborers can achieve through structural transformation. Furthermore, agriculture’s continued relevance to structural change in Sub-Saharan Africa is highlighted by the strong linkages observed between rural non-farm activities and primary agricultural production.

## 1 Introduction

Structural change is integral to economic development. In the development context, it refers both to the reallocation of labor from one low-productivity sector to another, higher-productivity sector, and to the economic growth resulting from this shift. Structural change is a dynamic process powered by several key features – productivity levels within sectors, productivity gaps between them, and the movement of labor from low productivity to high productivity sector(s). The larger the productivity gap between agriculture and other sectors, the larger the opportunity to achieve productivity growth as labor shifts across sectors. In poor economies, agriculture is typically the sector that employs the most people and uses labor least productively. Over time, cross-sector productivity gaps tend to shrink as labor shifts out of agriculture and returns to labor across sectors are equalized through factor markets (Timmer, 1988).

**Fig. 1:**
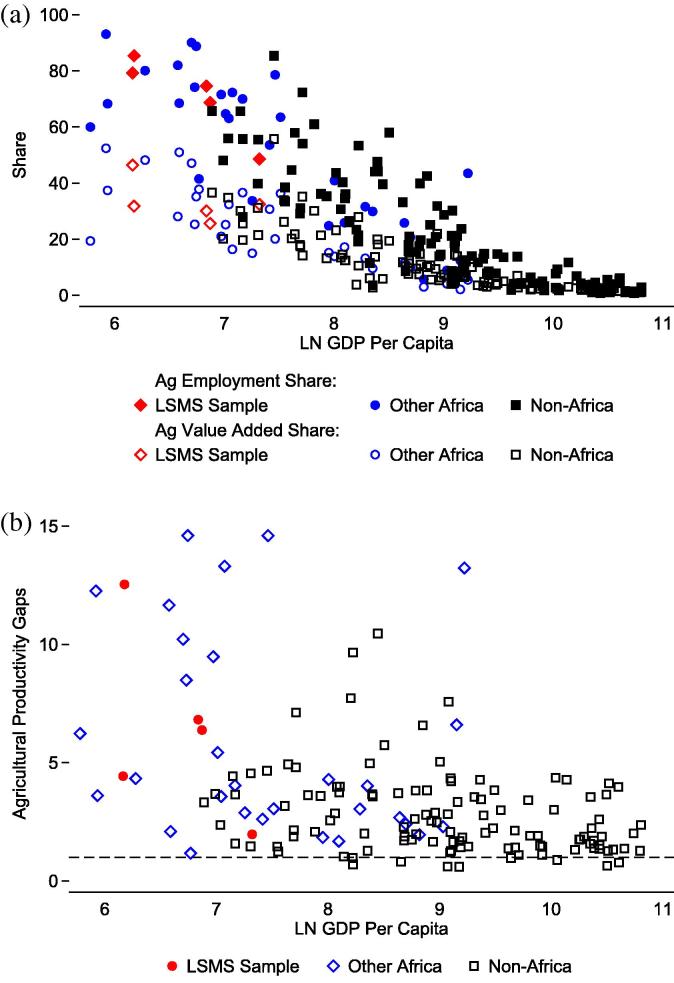
Figure (a) (top) shows a global cross-section of agricultural labor and employment shares graphed against a log transformation of each country’s per capita GDP. Figure (b) (bottom) shows agricultural labor productivity gaps graphed against the log of GDP per capita (Source: Gollin et al., 2014a, Gollin et al., 2014b). The horizontal dashed line represents inter-sectoral parity in labor productivity (value = 1).

The premise of higher returns to labor outside of agriculture is quite central to structural change. Are these productivity differentials really as high as national accounts data suggest? I use a new micro-level dataset to measure key structural change parameters – sector participation, time use, and labor productivity – from a micro perspective. This paper draws on the Integrated Surveys on Agriculture from the Living Standards Measurement Study group at the World Bank (LSMS-ISA datasets), which explicitly collect information about respondents’ time use across sectors. Particular attention is paid to farm labor, which is often neglected in large scale, multi-topic surveys because of the challenges involved in collecting detailed agricultural data. The analysis includes surveys from Ethiopia, Malawi, Tanzania and Uganda.^1^ The countries comprising the LSMS-ISA dataset exhibit considerable heterogeneity with respect to GDP per capita, agriculture’s share of the labor force and economy, and productivity gaps (Fig. 1).

Examining productivity gaps from a micro perspective is informative for several reasons. First, individuals and firm owners making labor allocation decisions in developing countries do so based on the micro incentives that they face. Second, micro datasets contain the variables required to address the validity of assumptions that underlie macro statistics. Third, micro datasets allow for productivity measures to be paired with relevant covariates of labor allocation decisions at the household and individual levels. This kind of micro perspective is largely absent from the literature about structural change in African economies. Demographic and Health Survey (DHS) datasets, also micro datasets, are sometimes used to calculate sector labor shares, as an alternative to measures based on population censuses or national accounts (e.g., McMillan and Harttgen, 2014, McMillan and Rodrik, 2011). While DHS surveys have very extensive coverage, they cannot be used to generate measures of labor supply beyond participation, nor can they be used to measure returns to sector participation.

I find that, in four Sub-Saharan African countries, the agricultural sector is not a bastion of low productivity but, rather, a large reservoir of underemployed workers. This result emerges when labor inputs are measured more carefully. Using the LSMS-ISA datasets, I replicate common patterns observed in macro statistics – that annual economic output per worker is lower in agriculture than in other sectors and that participation in agriculture is much higher than participation in other sectors. While national statistics suggest that workers in these four countries are 6 times as productive outside of agriculture as in it, I predict the number is closer to 3.4 times on average. This finding is consistent with those of Gollin et al. (2014b), who highlight sources of bias in national accounts measures that lead to under-estimating productivity in agriculture relative to other sectors.

After carefully examining labor inputs, I find that cross-sector productivity gaps observed in national accounts data reflect sectoral differences in employment levels rather than differences in returns per hour worked. Many workers are counted as agricultural because they spend at least some time working on farms. A striking pattern across household surveys is that agricultural workers work fewer hours per year – 700 hours per agricultural worker compared to 1850 hours per non-agricultural worker. Productivity in agriculture is a lot closer to productivity outside of it when one accounts for systematic differences in labor inputs. On a per-hour basis, labor is only 1.4 times as productive outside of agriculture.

These results suggest that the forces pulling labor into the industry and service sectors may be weaker than is commonly believed. It also casts doubt on the notion that agriculture is intrinsically less productive than other sectors. Because time inputs in agriculture are generally low, possibly due to biophysical constraints, participation outside of agriculture presents the opportunity to supply more hours of labor per year. It is important to better understand the reasons for low labor supply by agricultural workers in order to identify opportunities to increase annual output per agricultural worker.

## 2 Background

This paper focuses on Sub-Saharan Africa, the region with the lowest per capita incomes, largest shares of value added captured by agriculture, largest shares of the work force employed in agriculture, and lowest agricultural labor productivity (Fig.1a) (World Bank Group, 2014). According to national accounts data, labor in developing countries is 4.5 times more productive outside of agriculture than in it. In middle income countries, the ratio is 3.4, and in high income countries, it is 2.2. Within African countries, non-agricultural labor is 6 times more productive outside of agriculture than in it^2^ (Fig.1b) (Gollin et al., 2014b). Other recent studies confirm that large cross-sector productivity differentials persist in Sub-Saharan African countries (McMillan and Harttgen, 2014, Lele et al., 2013).

Labor productivity in an economy can be improved either within sectors (e.g., through technological gains and capital accumulation) or structurally (e.g., by shifting labor out of less-productive activities and into more-productive activities).^3^ During the 1990s, African labor entered agriculture rather than exiting it, thereby suppressing overall labor productivity growth (McMillan and Rodrik, 2011). Since 2000, labor productivity growth within agriculture has accelerated in Eastern and Southern Africa, and in Nigeria (Pardey, 2014, Block, 2013). When recent labor productivity growth is decomposed into within- and between-sector growth, labor exits from agriculture account for about half of recent overall labor productivity growth in Africa (McMillan and Harttgen, 2014, McMillan et al., 2014).

Understanding micro level cross-sector productivity differences, and how they relate to sector allocation decisions, is crucial for understanding the forces that power agricultural labor exits. If productivity gaps are indeed as large as African macro statistics suggest, then one must wonder why so much labor remains in rural areas and why rural income diversification remains so low (McMillan and Headey, 2014). One explanation is that, though households may face large productivity gaps, they are not able to diversify because of limited human capital, experience, or financial capital. It is also possible that differences in expected returns between sectors are offset by different levels of risk.

Alternatively, national accounts may mis-measure key components of the productivity equation, namely, labor inputs or returns per worker. After examining many of the assumptions used to measure agricultural labor productivity gaps from national accounts data, Gollin et al. (2014b) find a number of biases that inflate estimates of productivity gaps. These biases arise from the methods used to classify workers as agricultural or non-agricultural, the assumption that workers in each sector work an equal number of hours, and the assumption that workers from each sector have the same levels of human capital.^4^ Even after correcting for these biases, the authors find that large productivity gaps remain, with an average corrected productivity gap of 3.3 in Africa.

Another explanation for small micro gaps and large macro gaps is that micro gaps are truly smaller than macro gaps. The cross-sector gaps that households face will be smaller than those suggested by national accounts, should the differential returns to non-agriculture sector activities accrue to owners of capital rather than labor. In capital-intensive industries like mining, wage rates are likely to be much lower than average labor productivity in mining as per national accounts data (McMillan and Harttgen, 2014).

If there is systematic measurement bias across sectors, then productivity gaps calculated from national accounts data will be biased. This paper generates micro-based productivity measures in order to highlight the productivity gaps that households face and to inform the debate about productivity mis-measurement in national accounts data.

Consider the productivity gap between agriculture and services, decomposed into labor inputs (hours per year) and productivity (returns per unit of labor input):

$${\mathrm{GAP}}_{S}=\frac{\frac{Y_{S}}{N_{S}}}{\frac{Y_{A}}{N_{A}}}=\frac{\frac{Y_{s}}{H_{s}}*\frac{H_{s}}{N_{s}}}{\frac{Y_{A}}{H_{A}}*\frac{H_{A}}{N_{A}}}={\mathrm{PGAP}}_{S}*{\mathrm{EGAP}}_{S}$$

where Y_S_ refers to service sector output, N_S_ refers to the number of service sector workers, and H_S_ refers to the annual hourly input of a service sector worker. The A subscript refers to the agriculture sector. The gap in annual output per worker between the service and agriculture sectors can then be decomposed into a gap in productivity per hour worked across the sectors (PGAP_S_) and a gap in employment levels across the two sectors (EGAP_S_). If cross-sector productivity is equalized in terms of returns to hourly or daily labor at the margin, and labor productivity gaps are largely explained by cross-sector differences in labor inputs, then one can no longer argue that labor is grossly misallocated across sectors even when there is a large cross-sector gap in annual returns per worker. This has implications for the forces that drive labor exits – they relate to seeking fuller employment rather than climbing a per-hour productivity gradient.

Claims of underemployment in a smallholder sector have been common, historically. Lewis’ two-sector model was premised on “unlimited supplies of labor,” positing labor surplus in subsistence agriculture and also among casual laborers and those self-employed in petty trade (Lewis, 1954). Lewis also discusses “disguised unemployment,” whereby many family members supply labor to the household farm, but, should one of the household members be able to find work elsewhere, the same level of output could be maintained if the remaining household members increased their labor supply on the intensive margin. Surplus labor remains relevant, today, in the form of large reservoirs of developing country workers who engage in informal activities and part time work with irregular hours that is characterized by low returns to skill (Gollin, 2014).

Because agricultural labor shares are large in African countries, the potential gains from reallocating labor to higher-productivity sectors are also hypothesized to be large (McMillan and Headey, 2014). A large initial agricultural labor share, rising female education, rising commodity prices, good governance, and agricultural productivity growth all appear to be positively correlated with labor exits from agriculture (McMillan and Harttgen, 2014).

Though African countries seem to be following the same patterns of agricultural labor exits as those followed decades earlier in Asia and Latin America (McMillan and Harttgen, 2014), there are some important differences. The services sector, which is characterized by relatively low productivity in African countries, has been a primary recipient of labor exiting from agriculture (Rodrik, 2014). In other regions, industrialization has been core to the structural change process. High levels of informality in the industry and services sectors has lowered their average productivity and suppressed the gains to be exploited from agricultural exits. In Vietnam, important productivity gains resulted not only by shifting labor out of agriculture, but also by shifting labor from informal into formal, higher-productivity firms within the industry sector (McCaig and Pavcnik, 2013).

Growth in labor productivity, overall and within agriculture, has been a strong predictor of poverty reduction because of the important linkages between wages, household self-employment, and the real incomes of the poor. Though land productivity growth typically precedes labor productivity growth, the process of agricultural development is thought to begin when output per agricultural worker increases (Timmer, 1988). Agricultural labor productivity growth is particularly important because of the direct effects on the many workers who participate in the agricultural sector, and also because of its effects on growth in other sectors (De Janvry and Sadoulet, 2010, Christiaensen et al., 2011).^5^

Labor is one of several important factors in agricultural production, which also relies on land, capital, and other inputs. Where land and capital are scarce (e.g., due to high population pressure or high interest rates, respectively), labor is used more intensively in farming systems. In aggregate, agricultural labor productivity grew slower than agricultural land productivity between 1961 and 2010 in Africa, which implies that African agriculture has intensified with respect to labor (Pardey, 2014). However, Binswanger-Mkhize and Savastano (2014) find that, while population density has increased in rural areas across LSMS-ISA countries, there has been little evidence of Boserupian agricultural intensification with respect to cropping intensity, area farmed, or irrigation.

## 3 Data and variable construction

To examine labor productivity gaps from a micro-economic perspective, I generate labor productivity measures and other key variables from the Living Standards Measurement Survey – Integrated Surveys in Agriculture (LSMS-ISA) dataset. I draw on a cross-section of recent LSMS-ISA datasets available, comprised of the Ethiopia *Rural Socioeconomic Survey* (2013–14), the Malawi *Integrated Household Survey* (2010–11), the Tanzania *National Panel Survey* (2010–11), and the Uganda *National Panel Survey* (2010–11). LSMS-ISA surveys were implemented by each country’s national statistics office, with technical support from the World Bank Development Economics Research Group. These datasets are nationally representative, including urban and rural households regardless of occupation or sector of employment. Rural and urban areas are defined by each country’s statistics office.

Table 1 depicts the basic characteristics the datasets used in this study. It is worth emphasizing the novelty of LSMS-ISA datasets. Surveys of farming populations often collect detailed plot-level farm management information similar to the LSMS-ISA surveys, but they do not also include information on time use off the farm, and generally do not sample non-farming households. The multi-topic, multi-purpose LSMS-ISA questionnaire includes questions on labor market participation, labor inputs into household farm and non-farm enterprises, and returns to enterprises and labor market participation. They are also internationally comparable to some extent, allowing for cross-country comparisons.

Using the LSMS-ISA data, I construct individual level, annualized labor supply aggregates for three types of activities – household operated farm enterprises (farms), household operated nonfarm enterprises (NFEs), and wage labor market participation. Labor supply recall questions differ in the LSMS-ISA surveys by type of activity. Appendix A contains detailed information about the construction of all of the variables used in this analysis.

Wage labor supply variables are generated over a twelve month recall period from individuals’ reported number of months worked over the last year, typical number of weeks worked per month, and typical number of hours worked per week. In the agriculture modules of the surveys, labor inputs by individual household members are collected for each farm plot. These inputs are aggregated for each household member to generate the annual own farm labor supply variable. For non-farm enterprises, participation by household members is flagged at the firm level. NFE labor supply collection differs slightly from country to country, as detailed in Table A.3 in Appendix A.

Systematic measurement error in construction of labor supply variables is particularly concerning, should respondents recall different types of activities with different errors. Differences in recall period (through questionnaire design or timing of interview) or differences in recall ability for different activities (e.g., rare, “salient” events vs. common ones) can lead to differences in household responses (Beegle et al., 2012, Bound et al., 2001). The possibility of measurement error in the constructed labor supply aggregates is addressed in Section 4 of this paper.

Next, I construct aggregates of labor demanded by household operated farms and NFEs, which include hired labor in addition to labor supplied by family members. Of interest are both the number of firm workers and the total labor inputs supplied by workers to each firm. In the case of farm enterprises, we have a good measure of labor inputs, the number of household members who work on the farm, and the total number of hours worked by household members and hired workers. We do not, however, observe the number of employees hired. It is quite common for farm households to hire in some labor (between 30% and 94% of farms do it). In order to avoid under-estimating the total number of farm workers, I predict the number of hired workers by assuming that hired workers work the same hours as own farm workers. In the case of NFEs, we universally observe the number of hired workers but not the hours that they supply to the firm. Non-farm enterprises do not commonly hire workers. In all cases, fewer than 19% of households operating an enterprise hire in any workers.

Returns to labor market participation are comprised of the gross total wages received by wage workers, including in-kind payments (e.g., meals received) and gratuities. Costs of participating in wage labor markets are not measured so it is not possible to construct a net returns measure. The returns to operating a farm enterprise are based on net farm revenue, which is analogous with the “value added” concept that underlies national accounts data. The net value of farm output is derived from the Rural Income Generating Activities (RIGA) calculations and includes the value of own-consumed farm output as measured through the consumption module (Davis et al., 2010). For non-farm enterprises, reported enterprise profit is considered a more reliable measure of net firm revenue than a constructed measure based on gross revenues minus costs (de Mel et al., 2009). Where available, I construct the annualized firm level net revenue variable using reported profits. Otherwise, I use the household estimate of gross NFE revenue and subtract household estimated costs. To facilitate cross-country comparison, all measures of returns are converted to constant international dollars using the purchasing power parity conversion factor for private consumption from the World Bank’s World Development Indicators.

Using the labor supply variables and the returns variables, I construct average labor productivity variables. These are done separately for the three types of activities – wage labor, farms, and NFEs – as a simple ratio between returns to an activity and labor inputs into the activity. Two types of average labor productivity measures are constructed. The per-worker measure is based on output per worker per year. The per-hour measure is based on output per hour of labor supplied to each activity per year. Because we do not observe how many hours hired workers supply to NFEs, I am unable to generate per-hour productivity measures for these firms.

The next task involves generating sector level labor productivity measures, which aggregate, at the sector level, returns from and labor inputs to self-employment, wage employment, and farming. First, all activities are assigned to their respective sectors of the economy (i.e., agriculture, industry, or services). Following McMillan and Harttgen (2014), I group these into the general categories of agriculture (primary agricultural, livestock, and fishery and forestry production), industry (manufacturing, mining, construction, and public utilities), and services (wholesale and retail trade, transport and communication, finance and business services, and community, social, personal and government services). I generate sector level aggregates of labor supply and returns for each household. Farm activities are classified as agricultural. Wage labor and NFE activities are classified using the Industry Standard Industrial Classification (ISIC) codes provided with each activity’s description. An additional sector definition of “unknown” is used when individuals report jobs for which no description or sector code is available. These labor sources most likely occur in the agriculture sector, but I avoid assuming so.

The hourly agricultural labor supply aggregates do not include livestock and post-harvest labor. And the corresponding agricultural labor productivity measures do not include revenue from livestock in the numerator. In the per-person agricultural labor productivity measure, the numerator includes net livestock revenue (taken from the RIGA dataset), and the denominator includes workers who participate in livestock rearing. Table 2 presents a high level overview of the contents of each constructed productivity variable.

## 4 Corroborating macro and micro evidence

### 4.1 Sector labor shares

Often in the macro measures of sector productivity, individuals are constrained to one sector of participation, and it is assumed that individuals in each sector work the same number of hours and do not supply labor to secondary sectors. Usually, each sector’s labor inputs are assumed to be of the same skill and not adjusted for different levels of human capital. Initial examination of these assumptions using LSMS-ISA data suggests that they are indeed problematic and lead one to overestimate labor supplied to agriculture relative to other sectors, thereby artificially inflating estimates of the labor productivity gap between agriculture and other sectors (Gollin et al., 2014b).

**Fig. 2:**
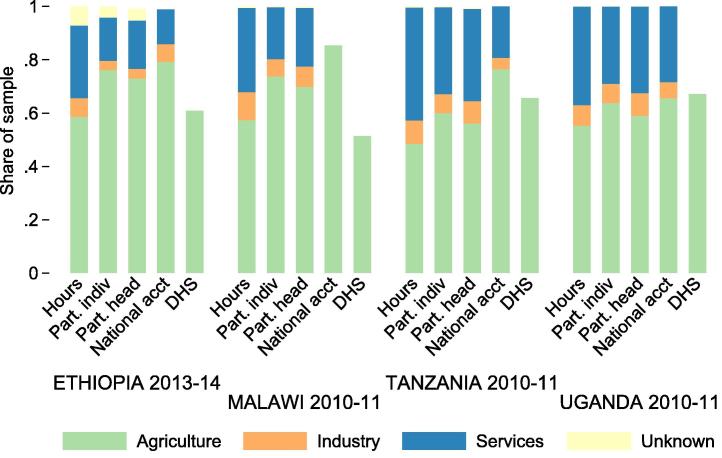
Comparison between different estimates of sector labor shares. The “Hours” measure is from variables generated using LSMS-ISA data. The “Part. indiv” measure is based on the primary occupation (most reported hours) of individuals in the dataset. The “Part. head” measure is based on the primary occupation of the household head. The “National account” measure is from the World Development Indicators database, and the “DHS” measure is based on DHS surveys, as described in the text.

Fig. 2 depicts three different measures of sector labor shares constructed using LSMS-ISA data along with two other commonly used measures – from national accounts and from Demographic and Health Surveys (DHS).^6^ The first column in Fig. 2 is based on the labor supplied by all adult individuals in the LSMS-ISA dataset.^7^ The second is based on the primary sector of each adult individual in the household, i.e., the sector to which each individual supplies the most hours.^8^ The third is based on the primary sector of the household head. This sub-sample includes individuals who reported positive hours worked in any sector.

Several patterns are common to all of the countries depicted in Fig. 2. First, agriculture is the dominant sector of participation across all data sources and aggregation methods, and participation in services is generally more common than participation in industry. Second, agricultural labor share estimates are slightly higher when they are based on all adult individuals in a household rather than just the household head. This suggests that household non-heads are more likely to work in agriculture than household heads. Third, hours-based agricultural labor shares are lower than participation-based shares, which is further explored below. Fourth, individual-based estimates of agricultural labor shares are lower than national-accounts measures in all countries. Fifth, the DHS-based measures of agricultural participation shares are quite a bit lower than the LSMS-based individual participation shares in Ethiopia and Malawi. This implies that, in these countries, DHS-based labor share estimates might under-estimate agricultural labor shares and therefore overestimate labor productivity in agriculture relative to other sectors. Since individuals self-identify their primary sector in DHS surveys, it is possible that respondents involved in multiple sectors are more likely to identify the non-agriculture occupation even though it accounts for a lower share of labor supplied.

Per-person productivity measures based on categorizing individuals by their primary sector of occupation implicitly ignore individuals’ contributions to secondary sectors. They also assume that participants in different sectors supply equal hours of labor. Both assumptions are problematic when individuals supply labor to secondary sectors, or when there are systematic cross-sector differences in hours supplied. Indeed, LSMS-ISA datasets suggest that both assumptions are violated.

**Fig. 3:**
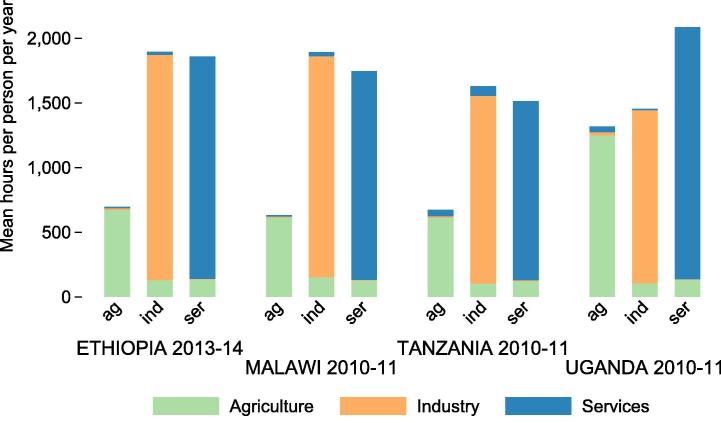
Average hours supplied by individuals to all sectors, categorized by each individual’s primary sector of participation.

Fig. 3 examines the one sector assumption, categorizing individuals by their primary sectors and depicting the average hours supplied to individuals’ primary as well as secondary sectors. The data imply that both the equal-hours and primary-sector assumptions are problematic. While those who are primarily categorized as agricultural laborers do not supply much labor to other sectors, workers who are primarily in industry or services sectors do supply labor to agriculture. Because secondary sectors are an important part of individuals’ labor supply, we likely underestimate labor supplied to agriculture by ignoring the labor supplied by individuals who participate in agriculture as a secondary activity, thus leading to an overestimation of labor productivity in agriculture relative to other sectors. Gollin et al.’s (2014b) analysis on secondary sector bias suggests that labor supply to non-agriculture by agriculture workers is greater than labor supply to agriculture by non-agriculture workers. These data indicate bias working in the opposite direction, with non-agricultural workers supplying more agricultural labor than agricultural workers supply to non-agriculture.

**Fig. 4:**
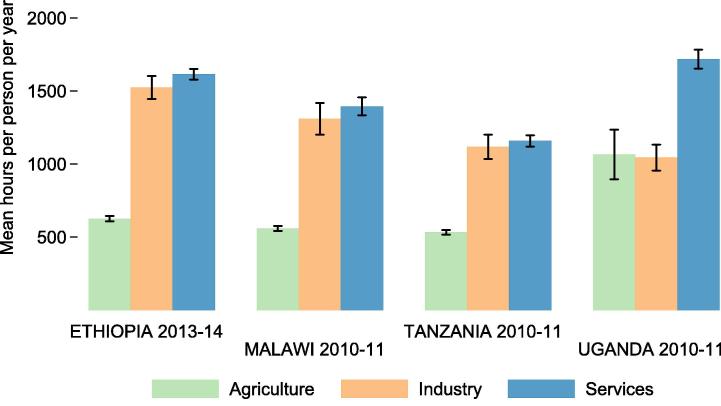
Average hours worked per year by sector participants. This sample includes all individuals between the ages of 16 and 65 who actively participate in the labor force. 95% confidence intervals for the mean are also depicted.

Violation of the equal hours assumption, on the other hand, leads to overestimation of agricultural labor inputs. Fig. 4 depicts the average hours worked in a sector by those who participate in it. Generally, those working in non-agricultural sectors supply significantly more hours than those working in agriculture. Gollin et al. (2014b) address the differences in hours supplied by agriculture and non-agriculture workers, using rural and urban distinctions where sector distinctions are not available. They find that, in poor countries, non-agricultural workers supply 1.3 times as many hours as agricultural workers to their respective sectors. This analysis confirms higher supply of labor to non-agriculture by non-agriculture workers than supply of labor to agriculture by agriculture workers, though our cross-sector differences in labor supply are large (between 2.3 and 2.5 in Malawi vs. Gollin’s 1.45, between 2.4 and 2.6 in Ethiopia, and between 2.1 and 2.2 in Tanzania). Our Uganda estimates, however, are smaller (between 1.0 and 1.6 vs. Gollin’s 2.3). These ratios are based on any form of sector participation (primary or secondary). When the sample is restricted to individuals who primarily participate in each sector, the ratios are quite similar.

Overall, the LSMS-ISA datasets suggest large gaps between hours supplied to agriculture and hours to industry and service sectors. By calculating sector labor inputs based on participation rather than hours worked, one over-estimates labor inputs in agriculture compared with other sectors.

The bars labeled “Hours” in Fig. 2 show the net effect of the equal hours assumption and the no-secondary-sector assumption on labor share measurement bias. In this case, the sources of bias offset each other. In all countries, agriculture’s share in labor is lower when an hour-based measure is used than when the LSMS-ISA participation-based measure is used. These results suggest that agricultural productivity may be underestimated relative to other sectors when participation-based labor shares are used. When the intensive margin of labor supply is controlled for by using hours-based labor share measures, estimates of agricultural productivity are relatively higher, and estimates of productivity gaps are smaller. This bias proves extremely important to any discussion about structural change in Sub-Saharan Africa.

### 4.2 Sector productivity gaps

**Fig. 5:**
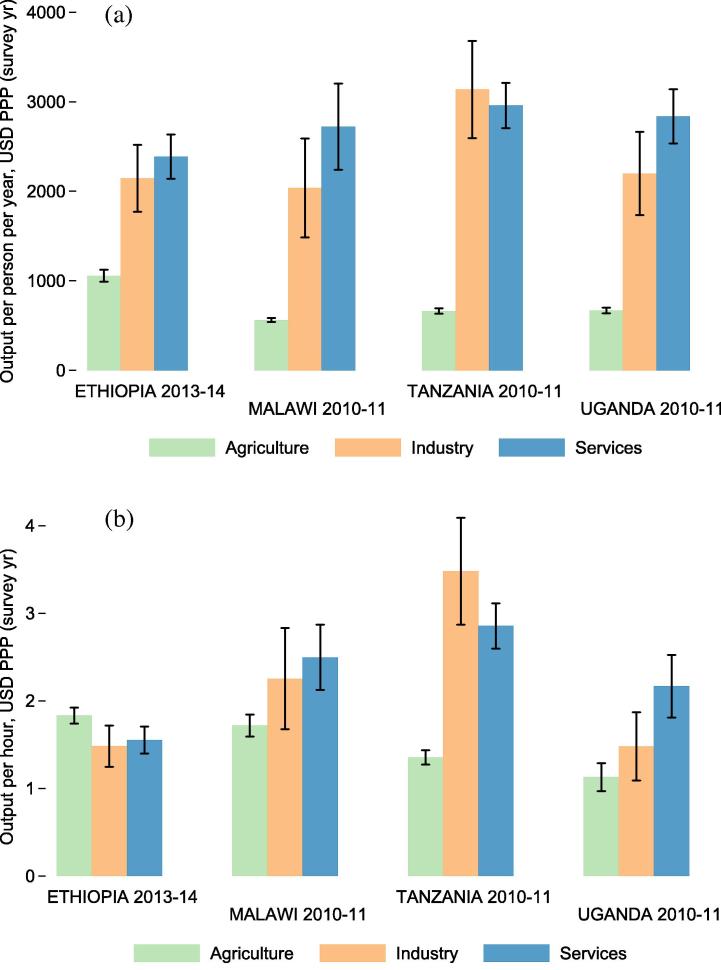
Productivity by sector. Figure (a) (top) shows annual value of output per sector primary participant per year. Figure (b) (bottom) shows output per hour worked per year.

Cross-sector productivity gaps calculated from the LSMS-ISA datasets are indicative of the average productivity differentials that households face when allocating their labor. Fig.5a depicts sector-level productivity measures with 95% confidence intervals in four LSMS-ISA countries, based on output per person per year. Output per worker per year is highest in the industry and service sectors, between $2000 and $3200 (USD ppp) per worker per year. Agricultural output per worker is between $560 and $1060 (USD ppp) per worker per year in all countries.

**Fig. 6:**
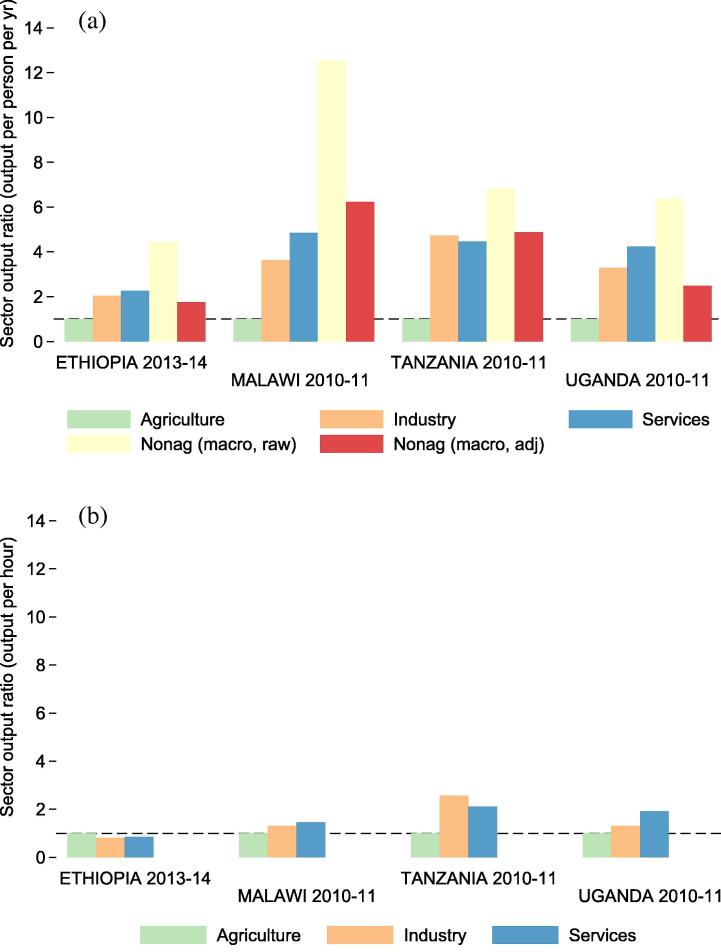
Productivity gaps by sector. Figure (a) (top) shows the ratio between productivity in each sector and agriculture based on per-person-per-year productivity measures. The fourth column depicts the raw productivity gaps between agriculture and non-agriculture as constructed using national accounts data, and the fifth column refers to adjusted gaps constructed by Gollin et al., 2014a, Gollin et al., 2014b. Figure (b) (bottom) shows the ratio between productivity in agriculture and in other sectors based on output per time input.

Fig.6a depicts micro-level productivity gaps (simple ratios between each sector’s productivity and productivity in the agricultural sector) along with national accounts based measures of productivity gaps, gathered for the purpose of comparison. These per worker measures of average labor productivity are not meant to replicate the output per worker measures generated from national accounts, which use different sampling approaches. Corporations are not sampled in the LSMS-ISA surveys, for example, so their activities are only detected through wages paid to workers hired by such firms. Should non-agriculture activities be more capital intensive, then capital ownership differences could explain why macro level productivity gaps are slightly larger than micro level gaps. Gaps in output per worker per year are smaller than national accounts gaps in all countries (Fig.6a).

Fig.5b shows sector level output per hour of labor worked in each sector. After adjusting for labor inputs (hours worked), returns per hour of labor supplied are between $1 and $3.50 (USD ppp) in all sectors. When considering time inputs in each sector, cross-sector gaps in productivity shrink considerably (Fig.6b). The hours-based gap measures are much smaller than the per-person-per-year gap measures in all countries. An hour worked outside of agriculture is 0.9 times as productive as an hour worked in agriculture in Ethiopia, 1.4 times as productive in Malawi, 2.1 times as productive in Tanzania, and 1.9 times as productive in Uganda.

Much of the productivity differences observed in national accounts statistics may then be attributable to differences in hours supplied by workers in each sector rather than differences in output produced per hour worked in each sector. Table 3 shows each country’s overall gap in output per worker per year, along with the two components of this gap – output per hour worked and hours worked per year. Employment gaps explain about half of overall micro level productivity gaps in Uganda, and a larger share in all other countries.

**Fig. 7:**
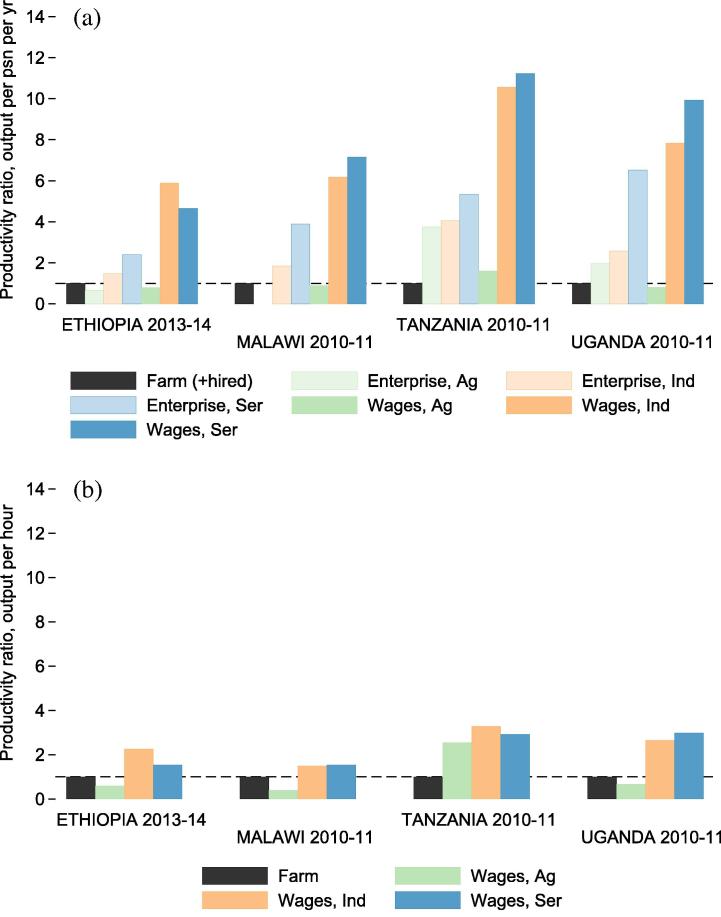
Productivity gaps by activity for all households (ratio between mean values for each activity). Figure (a) (top) depicts the ratio between mean farm labor productivity per person per year and the mean labor productivity of other activities (i.e. NFEs and wage labor in different sectors). Figure (b) (bottom) depicts per-hour productivity gaps for the same activities.

After further disaggregating returns to labor between self-employment and wage employment, it is clear that wage employment brings higher annual returns to participants than does self-employment. Fig. 7 depicts productivity gaps at the sector-activity level, with farming as the comparison activity. The sector-activities compared include household-operated farms, household-operated non-farm enterprises (NFEs) in all sectors, and wage labor in all sectors. Because hours or days of labor supplied by hired workers to NFEs are not collected anywhere besides Malawi, per-hour firm level productivity estimates are not included for NFEs. Wage labor returns should not be interpreted as measures of productivity, especially in the presence of market frictions, of which the evidence is strongly suggestive (Dillon and Barrett, 2014). They do offer a lower bound on the marginal revenue product of rented out labor, and they also provide a benchmark against which individuals in an economy can compare returns to self vs. own employment.

The sector-activity patterns depicted in Fig.7a are similar to the patterns observed at the sector level (Fig.6a). First, mean returns per participant per year are higher in industry and service sectors than in farming, whether the labor is supplied to NFEs or to wage employment. Within each sector, wage labor brings higher returns per worker per year than does self-employment. Wage laborers in the agricultural sector earn lower annual returns than industry and service sector laborers in all countries. The returns to agricultural wage labor are lower than the returns to own-farm labor in Ethiopia, Malawi and Uganda, and only slightly higher in Tanzania. At the hourly level, productivity gaps between farming and wage employment in industry and service sectors shrink considerably due to differences in labor supply between farm workers and non-agricultural wage laborers.

The existence of cross-sector gaps in output per worker per year suggests there are some forces enticing smallholder farmers into industry and service sector activities. Participation in industry and service sector activities may allow for fuller levels of employment in terms of hours of labor supplied per year. It is not possible, with cross-sectional data, to determine whether agricultural workers tend to work fewer hours because of constraints to labor supply or to labor demand. Biophysical and agronomic characteristics could limit the periods during the year in which farm labor can be used productively. In this case, it might not be possible for individuals to increase their agricultural sector returns by supplying more labor to their farms. Presumably, because labor supply is so low across households and countries, low demand for labor by agriculture is a key constraint. Agriculture’s role as a low entry barrier sector could help explain both high levels of participation in farming and low per-worker labor supply. Though individuals may aspire, and even attempt, to participate in non-farm activities, they may still return to farming as the sector that can basically guarantee employment. Labor transitions back into agriculture by individuals who had exited farming has occurred in Uganda (Christiaensen and Kaminski, 2015). Understanding what limits supply of and/or demand for labor in the agricultural sector is an important topic that is left for future research.

Within urban areas, self-employment in the service sector does not seem to serve as a sink for underemployment as does agriculture in rural areas. One might expect high rates of declaring self-employment due to possibly lower entry barriers than wage employment. Using the LSMS-ISA datasets, Nagler and Naudé (2014) show that self-employment participation correlates include wealth, credit access, and education. Self-employed workers in the industry and service sectors in urban areas tend to supply far more hours per year than do urban wage workers. The annual returns per worker to industry and service sector self-employment are much higher in urban areas than in rural areas, a finding consistent with Nagler and Naudé (2014). By assuming household firms do not hire in outside labor, one can estimate an upper bound on hourly returns to self-employment. These productivity estimates are very low, suggesting workers have a desire to supply labor even despite low returns.

## 5 Robustness of productivity gap measurement

Next, I turn to showing that these productivity gap measures are robust. I am concerned with both the measurement of labor inputs and the returns to labor. The first major concern is sensitivity of labor productivity measurement to survey timing. Labor supply varies seasonally and is elicited over discrete recall periods, raising the possibility that seasonal bias enters into labor productivity measurement. The second major concern arises from questionnaire design issues. Different types of labor supply are collected through different survey modules and elicited in different ways. The goal is to show that the key insights regarding sector participation, labor supply, and productivity are fairly robust to survey design. At the end of this section, I turn to measurement of the returns to labor, showing that the same patterns hold if consumption is used instead of income as a measure of returns to labor.

Annualized labor supply measures of participation and hours worked comprise the denominators of per-person-per-year and per-hour productivity measures, respectively. These aggregates are constructed from more detailed labor supply questions asked of respondents, such as the number of hours worked in the last week, or, in some cases, the number of hours worked in a typical week. One would expect these aggregates to move seasonally due to seasonal patterns in labor supply or a combination of seasonality of labor supply and recall bias in the case of a “typical week” recall approach.

**Fig. 8:**
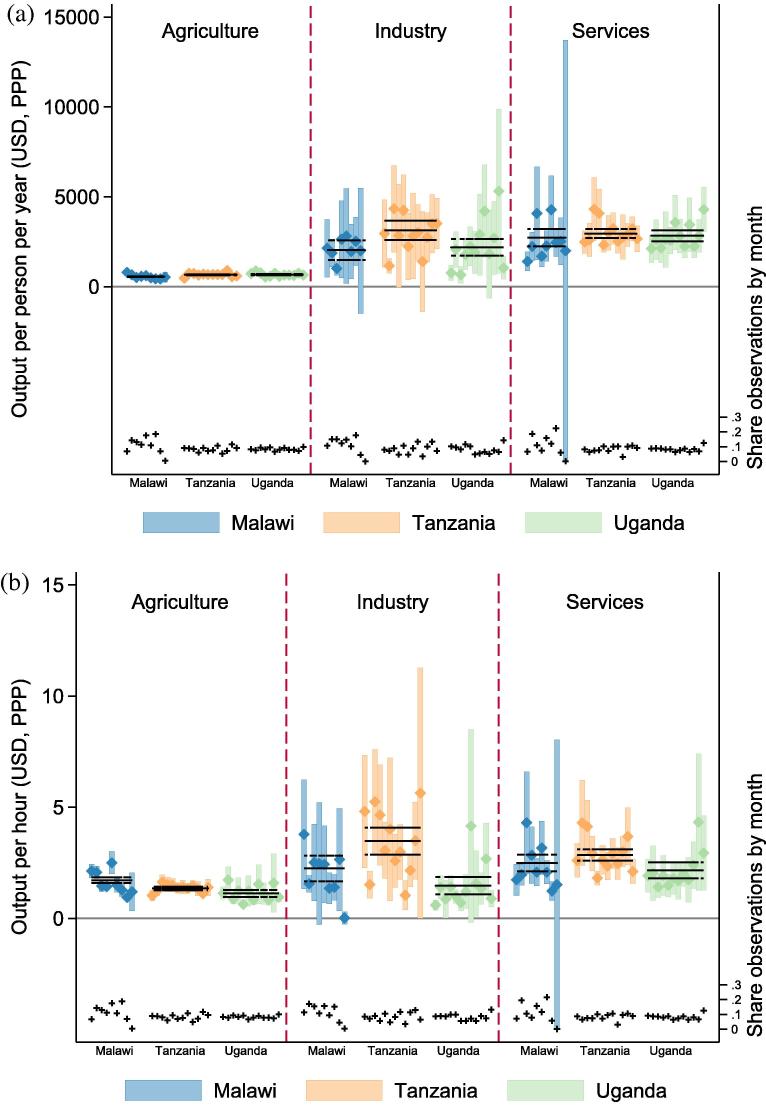
Figure (a) (top) shows average annualized output per worker per year by month of household interview (and the 95% confidence interval for each productivity measure). The horizontal line shows the annual mean for each productivity measure, with the dashed lines above and below depicting their 95% confidence intervals. And the share of observations per month is plotted at the bottom of the figure along the right hand axis. Figure (b) (bottom) shows sectoral output per hour of labor supplied, along with the annual mean for output per hour worked.

I demonstrate how the per-worker-per-year and per-hour labor productivity measures vary by month of survey visit in Fig.8.^9^ Each diamond represents a monthly mean productivity measure, and the bar it sits within depicts 95% confidence intervals for the mean. The horizontal solid line represents the annual survey-weighted average for the survey, along with dashed lines above and below representing its 95% confidence intervals. If more surveys are conducted during high or low productivity times within the year, then annual productivity aggregates would be biased. This is especially concerning if different sectors have different seasonality patterns within a country. According to Fig. 8, there are some months with especially high or low productivity measures, but there does not seem to be a major pattern of over- or under-representing these months.

In order to address concerns that survey timing is somehow correlated with seasonal productivity patterns, I generate new population-month weights to create annually representative measures of per-person-per-year and per-hour productivity for each sector. Using the weights, I also generate annually representative measures of the different components of labor supply. On the extensive margin, this includes participation on an annual basis. On the intensive margin, this includes participation in the last week conditional on participation in the last year and hours of labor supplied per week. I conduct a *t*-test for difference between the survey weighted means depicted in Section 3 and these survey-month weighted means. In Uganda and Tanzania, I cannot reject the null hypothesis of equal means between survey weighted and seasonally corrected measures of productivity or labor supply. In Malawi, there is evidence that, by not correcting for seasonality, agricultural labor supply is under-estimated and wage labor supply is over-estimated. If these biases were to be removed, per person productivity gaps would be the same but per hour productivity gaps would be slightly larger. The effect is small in magnitude (7% of the uncorrected per-hour productivity measure), and the difference is significant at the 10% level. This analysis suggests that seasonal bias due to survey timing does not bias the key labor supply or productivity variables.

Because labor supply variables for different activities are constructed from different types of survey questions, there is concern that differences in labor supply across activities could arise from different survey recall approaches rather than actual labor supply differences. In particular, downward bias in the measurement of agricultural labor supply or upward bias in self-employment or wage employment labor supply would undermine the agricultural underemployment findings. In a recent methodological experiment designed to compare different approaches to measuring farm labor inputs, Arthi et al. (2016) find that end-of-season plot based recall measures inflate farm labor supply considerably. The labor supply aggregates generated using the LSMS-ISA approach are about twice as large as labor supply aggregates generated by weekly eliciting the data from respondents in person or over the telephone, which is considered to be a more accurate approach. The LSMS-ISA approach also generated aggregates that were larger than those generated using a standard, stylized seasonal recall of days worked, without collecting plot specific information. These findings suggest that, given survey design, labor supply for smallholders is likely to be over-estimated rather than under-estimated. If this is the case, then underemployment within agriculture would likely explain an even larger share of productivity gaps. There has been little research on recall bias for wage and self-employment labor in the developing country context. However, the experimental evidence suggests that, in the LSMS-ISA surveys, farm labor aggregates are higher than they would be if measured using a stylized seasonal recall approach, which is comparable to the approach used to gather wage and self-employment data in the LSMS-ISA datasets.

Sector productivity gap estimates are sensitive to prospective measurement error of labor returns – farm and firm revenue and wage earnings. I use an alternate measure of returns to labor, household consumption, to ensure that the measurement of productivity gaps is robust to measurement of returns. Consumption can be thought of as household profits after participation costs (for wage labor) and production costs (for firms), assuming no savings or dis-savings. Because households who face stochastic income generally smooth their consumption from year to year, consumption can be a good measure of permanent income (Bhalla, 1978). It is a central focus of LSMS-ISA surveys to generate consumption aggregates, so this variable plays to the strengths of the data. Consumption aggregates are generated by each country’s statistics office and released with the datasets. They include cash expenditures as well as the imputed value of items that are produced and consumed by the household, such as agricultural goods.

**Fig. 9:**
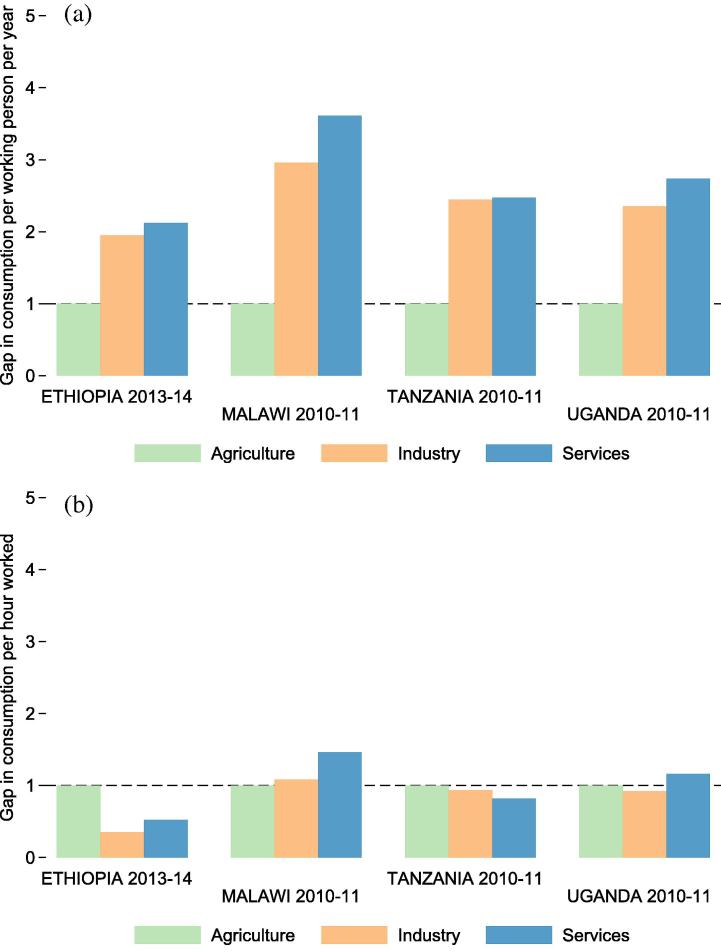
Consumption gaps by sector. Figure (a) (top) shows the ratio between consumption based on expenditures per working household member per year between households primarily participating in agriculture and those primarily participating in industry, services and ”unknown” sectors, respectively. Figure (b) (bottom) shows the ratio between consumption per hour of labor supplied by the household for households primarily participating in agriculture vs. in other sectors.

The consumption gap estimates are a ratio in annual per-worker consumption between households participating primarily in agriculture and those participating primarily in industry and services, respectively (Fig.9a). I also create an analogous per-hour measure, which is based on consumption per hour of labor supplied by households, including labor supply to secondary sectors. Households are classified by their primary sector of participation. The per-hour measures are shown in Fig.9b. These consumption gaps are fairly similar across countries, and are quite similar in magnitude to per-person-per-year productivity gaps. Households primarily in the industry sector consume 2–3 times more per capita per year than agricultural households. Households primarily in the services sector consume 2–4 times more per capita per year than agricultural households. As with productivity gaps, consumption gaps also disappear almost entirely when they are expressed per hour of labor supplied by each household. This suggests that differences in consumption across sectors (as with differences in returns to sector participation) can be explained in large part by differences in hours worked across sectors.

## 6 Exploring the non-farm economy

It is important to understand not only sectors of employment, but also the modalities by which workers supply their labor. Because of growth linkages between agriculture and non-agriculture, the specific types of activities to which workers supply labor can inform the scope for growth linkages between different sectors of the economy.

Recent micro evidence suggests that, while non-agricultural sources of income bring the highest returns across the welfare distribution, the majority of households in African rural areas remain specialized in agricultural income earning activities (Davis et al., 2014). After controlling for per capital income, though, households in Sub-Saharan Africa have similar diversification levels as households in other regions of the world.

A close examination of the non-farm activities in which households are involved suggests some clear patterns across countries. Workers outside of agriculture are more educated, younger, and less female than agricultural workers. Rural non-farm activities tend to be closely related to agriculture, with strong producer and consumer linkages.

### 6.1 Household and worker characteristics

It is important to explore any systematic differences in characteristics of sector participants, so that they can be taken into account when interpreting sector labor supply data generated from national accounts. Indeed, the macro-economic literature is concerned with systematic differences in human capital across sectors and the implications for bias in productivity measures (Vollrath, 2014).

Industry and service sector workers tend to be younger, on average, than agricultural workers. In all countries, the agricultural work force contains a larger share of women than men, while industry and service sector work forces contain more men than women. Palacios-Lopez et al. (2015) provide analysis of gender share of agricultural labor supply using LSMS-ISA data, pooling own and hired farm labor supply by gender. They find that women do not necessarily contribute a larger share of agricultural labor, in terms of person-days, than do men. The average years of education completed tend to be highest for services sector workers and lowest for agricultural sector workers. These educational differences point to possible systematic cross-sector skills differences. Individuals who do not supply labor to any sector are younger and more female, on average, than agricultural workers.

**Fig. 10:**
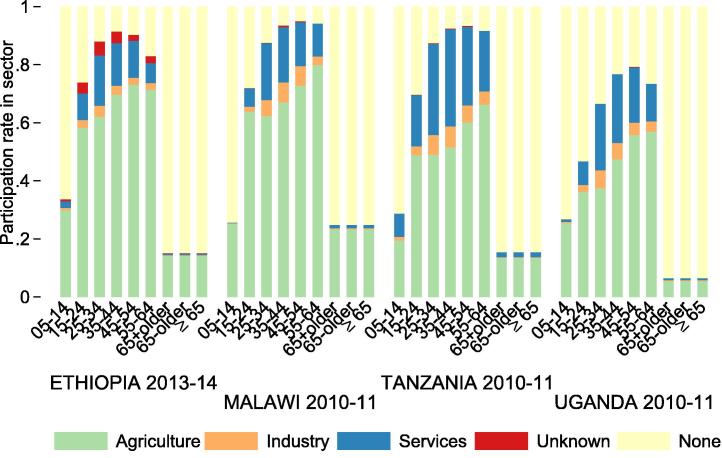
Primary sector participation rates across age cohorts.

Fig. 10 depicts the changing primary sector of workers across all major age cohorts. Youth (ages 15–24) have lower participation in economic activities than do young adults (ages 25–34). Economically active youth supply larger shares of labor to agriculture (compared to industry and services) than do economically active young adults. Despite these differences, labor shares are robust to the specification of the “adulthood” threshold at age 25 rather than age 15.^10^

Table 4a, Table 4b summarizes individuals’ participation in self and wage employment activities by sector, describing participation rates and basic characteristics of participants in both rural (Table 4a) and urban (Table 4b) populations. The tables summarize all individuals who participate in self and wage employment in each sector, not just those who primarily participate. Davis et al. (2014) generate household level estimates of participation in wage and self-employment by rural LSMS-ISA households.

Participation in self-employment is more common than wage labor participation in all countries, with 74–89% of rural adults participating in farming. Agricultural wage labor participation is less common than farming, with fewer than 15% of rural adults participating in Ethiopia, Tanzania and Uganda, and 33% in Malawi. In all countries, the average agricultural wage laborer is much more likely to be male than the average farm worker. Agricultural wage workers have more education, on average, than farm workers in Ethiopia and Malawi, and less in Tanzania and Uganda.

Behind agriculture, the services sector has the next highest overall participation rate. And in urban areas, the services sector is the most important. Workers are more likely to participate in the services sector through self-employment than wage employment in both rural and urban areas, except in urban Ethiopia where wage employment is higher than self-employment. Wage labor participants in the services sector are more likely to be male and to have higher education levels than self-employed service sector participants. Both wage and services sector participants supply similar numbers of hours per year except in Tanzania, where service sector self-employed workers supply far fewer hours than do wage laborers.

Within the industry sector, rural individuals typically are more likely to participate as self-employed rather than wage workers. Participation as either self-employed or wage workers is below 6.5% everywhere except Malawi, where one third of rural adults participate in industry sector self-employment.^11^ In rural areas, industry wage laborers are very strongly male, while industry self-employed workers are mostly female. Urban participation in industry sector activities is slightly higher than is rural participation everywhere except Malawi.

**Fig. 11:**
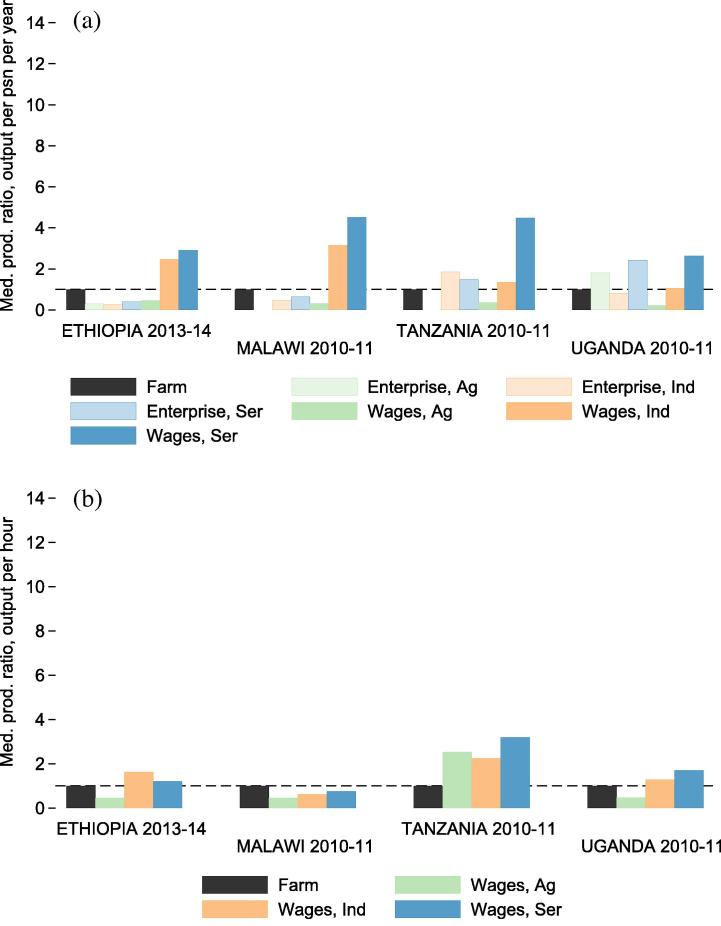
Conditional productivity gaps by activity for all households (median). Figure (a) (top) depicts the median of intra-household productivity ratios between farming and other activities, where productivity is defined as output per worker per year. Figure (b) (bottom) depicts the median of per hour intra-household productivity ratios. This analysis is based only on households that participate in farming and another activity.

There is always concern that differences in productivity may reflect observed and unobserved differences in the households and individuals participating in the activities rather than inherent differences in the economic productivity of the activities themselves. In an attempt to control for the effects of household level selection on productivity gap measurement, I generate within-household sector-activity productivity gap measures for households that participate in multiple activities. For households that participate both in farming and another activity, these reflect the ratio within the household between returns to the non-farm activity and farming. Fig. 11 depicts the median of intra-household productivity gaps. The conditional productivity gap for service sector wages, for example, is based on a comparison between annual returns per participant to farming and service sector wage labor within households who participate in both activities.

The conditional sector-activity gaps depicted in Fig. 9 are considerably smaller than the unconditional gaps depicted in Fig. 7. The fact that within-household gaps are so much smaller than between-household gaps suggests that heterogeneity in household characteristics between activity-sector participants could partly explain between-household productivity gaps. The observed small magnitude of intra-household gaps also suggests that structural barriers to improved household productivity that span across sectors may constrain households’ opportunities to raise their productivity levels. Such structural barriers to improved household productivity, including but not limited to the difficulties of accumulating human capital, would limit the opportunity to achieve productivity growth by shifting labor out of agriculture, even though productivity appears higher outside of agriculture on a per capita basis.

### 6.2 Farm and non-farm linkages

Table 5a, Table 5b breaks down non-farm self and wage employment activities into a more granular list of sectors. For the self-employment columns, the total number of households in the dataset is provided, along with the number of households that operate at least one non-farm enterprise, and the total number of firms present in the dataset. This final number is larger because some households operate more than one firm. These firms are then categorized by ten sub-sectors of the economy. In the non-farm wage employment columns, the total number of individuals of working age is listed, along with the total number who participate in wage labor, and the total number of jobs reported in the dataset. Again, because some individuals have more than one source of wage-earning income, the number of wage earning jobs is larger than the number of wage market participants. Industry sector activities are divided into mining, manufacturing, electricity and utilities, and construction. Service sector activities are broken into commerce, transport and communication, general services, and finance. Summaries of activities are provided separately for rural and urban areas (Table 5a, Table 5b, respectively). Many of the activities do not occur, or occur only once, in each sample.

Next, I use respondents’ free descriptions of their self-employment and wage employment activities, along with the detailed industry codes assigned by enumerators, to examine carefully the kinds of non-farm activities in which respondents are involved. Within each sub-sector, I use text analysis to identify the words that most commonly appear in respondents’ descriptions (Laver et al., 2003). We do not observe the level of formality associated with household firms and wage-earning jobs because there is not enough comparability across survey questionnaires to describe formality of employment arrangements and/or firm registration.

In rural areas of all four countries, agricultural wage labor is the largest category of wage employment. In Ethiopia, around 60% of wage employment occurs as casual or informal labor for which no sector information or job description was collected. Most likely, this labor is supplied to the agriculture sector.^12^ Based on text analysis of the descriptions provided, most agricultural jobs involve casual labor on farms for food or cash crop production, or they involve livestock tending, hunting, fishing, and collection of forestry products, such as fuel wood. Agricultural sector non-farm self-employment, which is not common, also tends to involve production of livestock, fishery, or forestry products.

Within the industry sector, mining does not play an important role in rural or urban areas of any of the datasets we analyze. Manufacturing accounts for between 13% and 38% of rural NFEs, with the smallest share in Tanzania and the largest in Malawi. However, only 1–9% of wage-earning jobs occur in manufacturing. According to text analysis, manufacturing NFEs focus heavily on elementary activities such as brewing alcoholic beverages, charcoal production, milling grains, butchering and other agricultural processing, baking and other value addition activities, and the production of household goods, clothing, and other handicrafts. Manufacturing wage jobs are similar, with a focus on agri-processing for cash crops, timber, and textiles, as well as the manufacturing of bricks and other building materials. Construction accounts for between 2% and 6% of rural wage jobs and between 5% and 9% of urban wage jobs but fewer than 2% of NFEs. Construction wage employment, according to text analysis, typically involves working as a laborer on a building or road construction site.

Individuals and households who participate in the industry sector are involved mainly in manufacturing activities that have strong links with primary agricultural production. Industry sector participants contribute to manufacturing raw agricultural materials into typically non-tradable goods meant for local consumption. These patterns suggest strong links between rural industry-sector activities and agriculture. In rural areas, the manufacturing industry stands to gain from productivity growth in agriculture, and rural manufacturing workers are poised to benefit from demand spurred by rising agricultural incomes in rural areas. Because the manufacturing activities reported in these surveys are so closely linked with agriculture, one would not expect to see expansion of rural industry sector activities independently, without any agricultural growth. These classic Mellor-Johnston linkages are quite prominently featured in rural households’ economic activities.

Commerce is the dominant focus of self-employment in the services sector, while jobs tend to involve general services provision. Commerce comprises between 26% and 66% of both rural and urban firms. These are involved in activities such as the wholesale and retail trade of fruits and vegetables, other food items, charcoal, second hand goods, and other household goods. Commerce accounts for up to 20% of wage earning in urban areas of Tanzania, but the share is more often closer to 5–10% in urban areas, and lower in rural areas. Commerce wage earners are most commonly sales clerks and store attendants. The general services category is the most important for wage employment, accounting for 42–45% of urban jobs across all countries, and 12–31% of rural wage earning jobs. These wage workers include teachers, health, social and religious workers, public administrators, technicians, domestic service providers, as well as restaurant, hotel, and tourism employees. General services account for a smaller share of firms than of wage jobs. The most common firm descriptions include restaurants, caterers, bars, hotels, professional service providers, and repair shops. The transport sub-sector accounts for a small share of self and wage employment everywhere. Transport activities tend to focus on transportation services provided by bicycle, taxi, bus, or vehicle. Finance and real estate are almost nonexistent in rural areas and account for 1–3% of urban wage earning jobs, which are most commonly administrative in nature.

Buying and selling agricultural products comprises a large share of commerce activity, with respect to both self and wage employment. As with the industry sector, the services sector activities in which rural households participate are non-tradable in nature, and very focused on local consumers. Because these service sector activities serve local consumers whose incomes are dominated by agriculture-sector activities, the Mellor-Johnston linkages are again quite prominent. One would expect agricultural productivity growth to spur demand for increased local service sector labor. Given the nature of service sector activities, it would be hard to imagine strong growth in the services sector absent agricultural growth.

## 7 Conclusion

Micro level cross-sector labor productivity gaps are smaller than those generated using national accounts data. Inter-sectoral differences in annual earnings per worker arise from differences in employment volume (hours per worker of labor supplied) rather than wages or productivity per hour of labor supplied. At least half of these per-worker productivity gaps can be explained by differences in hours worked across sectors. The tendency is for individuals participating in agriculture to supply fewer hours to agriculture, on average, than individuals participating in other sectors. Returns to an hour of labor supplied outside of agriculture are about 1.4 times as high as returns to an hour of agricultural labor, on average, in the four countries analyzed.

Generally, the micro evidence seems consistent with the idea that there is some scope for achieving productivity gains by shifting labor from agriculture to industry or services. Households expect industry and service sector wage workers to earn higher returns per year than farm workers. Self-employment brings higher annual returns to participants than farming but lower than wage employment. Since micro gaps are smaller than macro gaps, workers, who are the owners of labor, may not stand to reap the large benefits of labor exiting agriculture that are expected in the economy as a whole (should national accounts data indeed reflect true economy-wide productivity gaps). Small per-worker-per-year micro gaps also suggest that agriculture-sector workers do not feel as strong a “pull” from industry and services as one might expect based on national accounts data. Small per-hour gaps do not undermine agriculture’s role in structural transformation. Despite low per-hour gaps in agriculture, it appears that workers have an excess of labor that could be absorbed productively in other sectors. This requires growth in demand for labor within or outside of agriculture.

Though underemployment in agriculture has been observed in the developing country context, it is not a well understood phenomenon. Widespread underemployment could erode the benefits of using agricultural labor more productively. The existence of large employment gaps across sectors raises the question of what limits the supply of hours in agriculture and what role technology, infrastructure and policies might play in addressing agricultural underemployment. Smallholders could be operating at high levels of technical efficiency, yet face environmental production constraints, such as limiting in-season rainfall for rainfed crops (Sherlund et al., 2002, Schultz, 1964). In this case, there could be scope to smooth labor demand with water control infrastructure and management practices. Demand for agricultural labor could be constrained due to the time-sensitive nature of agricultural tasks, such as land preparation, planting, weeding, and harvest. If certain time-sensitive tasks create labor supply bottlenecks, then interventions to address these bottlenecks, such as mechanization, could generate productivity gains. Mechanization has been very limited in LSMS-ISA countries (Sheahan and Barrett, 2014), though this demand could reflect frictions in capital markets.

Barriers to participation in non-farm self or wage employment could limit labor supply outside of agriculture by underemployed agricultural workers (Barrett et al., 2001, Rodrik, 2014). These could arise from constraints to accumulating human capital, or limited opportunities for off-farm employment. The evidence suggests that individuals and households may indeed face barriers to participation in non-agriculture activities. Workers who primarily participate in the industry and service sectors tend to also participate in agriculture, while the reverse is not true of workers who are primarily agricultural. Service sector participants, in particular, tend to have higher education levels than workers in other sectors. Some households may face structural barriers to labor productivity growth that span across multiple sectors. The small size of conditional gaps (within-household gaps faced by participants in multiple sectors) relative to unconditional gaps (pooled, cross-sector gaps) suggests that selection effects into non-agriculture activities contribute to cross-sector productivity differentials. Households who are unable to diversify might face even smaller productivity gains outside of agriculture than those who are, further eroding the benefits of structural reallocation of labor.

Overall, the analysis emphasizes agriculture’s key role in Sub-Saharan African economies, while also raising questions about agricultural employment gaps, their determinants, and how they shape the opportunity to achieve economy-wide labor productivity growth. A between-sector gradient in annual output per worker remains to be exploited. Improving annual output per worker within agriculture, the highest participation sector by far, requires a better understanding of labor demand by smallholder farmers.

Agriculture, and specifically the operation of household farms, remains a dominant economic activity in rural areas of Sub-Saharan Africa. And, furthermore, much of the labor supplied to industry and services sectors involves the processing and trading of agricultural and other primary goods for consumers whose incomes are dominated by agriculture-sector activities. Furthermore, the non-farm activities in which rural households are involved, across countries, are incredibly closely linked with agriculture. These strong links highlight additional benefits to achieving agricultural productivity growth, which can increase the supply of raw materials for manufacturing and increase the demand for non-tradable goods and services. These linkages are also sobering as, apart from agriculture, no engine for rural economic growth is apparent.

## Figures and Tables

**Table A.1:** Wage labor variable construction notes.

	Ethiopia	Malawi	Tanzania	Uganda
	2013–14	2010–11	2010–11	2010–11
*Job tracking*	Up to two jobs, Productive Safety Net Program (PSNP) labor, and casual labor	Up to two jobs and casual (*ganyu)* labor	Up to two jobs	Up to 4 activities including jobs, own farm and NFE (2 most important in past 7 days and up to 2 more if they are more important over last year)
*Time use aggregate*	For jobs: months last year; typical weeks/month worked; typical hours/week worked. For casual labor (and PSNP in Ethiopia): days/last year (hours per day assumed)		Months last year; typical weeks/month worked; hours last week	Months/year; weeks/typical month worked; hours last week
*Cleaning (time)*	Assume unreported time observation = 0. Truncate unreasonably large values (e.g., >16 h per day or >7 days per week)			
*Sector in-formation*	Observed for jobs. PSNP is classified as ag. Casual labor is classified as “unknown”	Observed for jobs. Casual (*ganyu*) labor is classified as ag	Observed for both jobs	Observed for all activities (for 4th activity sector is observed but not whether it is a job, family farm, or NFE)
*Wage rate*	Earnings reported per pay period (for jobs) and annually (for casual and PSNP labor)	Earnings reported per pay period (jobs) and per day (casual labor)	Earnings reported per pay period	Earnings reported per pay period (lots of missing pay period observations)
*Cleaning (wages)*	Winsorize hourly wage rate (p = 0.01) and reconstruct annual returns with imputed wage			
*Annual returns*	For each job: cleaned wage rate ∗ annual hours			

**Table A.2:** Farm enterprise variable construction notes (for households involved in crop production).

	Ethiopia	Malawi	Tanzania	Uganda
	2013–14	2010–11	2010–11	2010–11
*Seasons included*	Only main (*meher*) season	Rainy and dry seasons	Rainy and dry seasons	First and second seasons
*Farm tasks included*	Post planting (land prep, planting, ridging, weeding, fertilizer application); harvest	Land prep/planting, pre-harvest; harvest	Land prep; weeding; post planting; harvest	All tasks lumped together
*Participation (indiv)*	Up to 6 hh members identified per task per plot/task combo	Up to 4 hh members per plot/task combo	Up to 6 individuals per plot/task combo	Up to 3 individuals per subplot
*Time use aggregate (indiv)*	For each indiv/plot/task: days/week (assume 7 h/day), weeks/season	For each indiv/plot/task: days/week (assume 7 h/day), weeks/season	For each indiv/plot/task: days per season (assume 7 h/day)	For each sub-plot: days per season (assume 7 h/day, assume equal labor division b/w individuals listed)
*Cleaning (time)*	Truncate unreasonably large values (e.g., an individual works more days than the length of the season)			
*Hired labor*	For each plot/task: male, female and child hired laborer person-days	For each plot: male, female and child hired laborer person-days for pre-harvest and harvest periods	For each plot/task: male, female and child hired laborer person-days	For each plot: male, female and child hired laborer person-days
*Exchange labor*	For each plot/task: male, female and child exchange laborer person-days	For each plot: male, female and child exchange laborer person-days	Not in survey	Not in survey
*Farm labor inputs (hours)*	Own farm labor + hired and exchange labor	Own farm labor + hired and exchange labor	Own farm labor + hired labor	Own farm labor + hired labor
	In all countries, days of household and hired labor are converted to hours (7/day). Labor inputs supplied by children (age 5–14) are assigned a weight of 0.5			
*Farm labor inputs (people)*	Number of family members with positive hours working on farm (no count of hired or exchange workers available). Children (age 5–14) are assigned a weight of 0.5			
*Annual returns*	Net farm returns are taken from RIGA (see Section 2 of paper). Quantity of auto-consumed farm production is estimated using the consumption module of the survey. Net returns are truncated at zero			

**Table A.3:** Non-farm enterprise (NFE) variable construction notes.

	Ethiopia	Malawi	Tanzania	Uganda
	2013–14	2010–11	2010–11	2010–11
*Participation (indiv)*	HH indicates operation of NFE; up to 5 HH members can be listed as workers	HH indicates operation of NFE; up to 4 HH members can be listed as workers	Indiv indicates whether he/she is involved in self employed activities	HH indicates operation of NFE; up to 5 HH members can be listed as workers
*Time use aggregate (indiv)*	Predicted for non-reporting households using NFE months in operation and median 7-day recall	For each HH member listed as NFE worker: months/last year; typical days/month worked; typical hours/day worked	Predicted for non-reporting households using NFE months in operation and median 7-day recall	Individuals report NFE employment in labor module (months/last year; typical weeks/month; hours/last week)
*Cleaning (time)*	Truncate unreasonably large values (e.g., >16 h per person per day)			
*Sector information*	Industry code provided in NFE module	Industry code provided in NFE module	Industry code provided in labor module	Industry codes missing for 2/3 of NFEs. They are predicted using industry coding from labor module
*Hired labor*	Number employees working for NFE (past 12 months)	Number of male, female and child employees (and hours worked) in a typical month of operation	& Number employees working for NFE (past month)	Number employees working for NFE (past month)
*Firm labor inputs*	Can recover number of workers (own and hired), not hours worked	Can recover number of workers and hours worked	Can recover number of workers (own and hired), not hours worked	Can recover number of workers (own and hired), not hours worked
*Annual returns*	Net firm revenues (reported gross sales minus reported costs)	Reported net firm revenues	Net firm revenues (reported gross sales minus reported costs)	Net firm revenues (reported gross sales minus reported costs)
*Cleaning (returns)*	Reported revenue and cost variables are winsorized (p = 0.01). Net revenue variable is truncated at 0			

**Table 1:** Dataset characteristics.

	Ethiopia	Malawi	Tanzania	Uganda
	2013–14	2010–11	2010–11	2010–11
Households in sample	5262	3247	3846	2633
Urban households (share)	0.173	0.245	0.307	0.163
Household size	4.854	4.672	5.091	4.888
(sd)	(2.31)	(2.25)	(2.93)	(2.68)
Household size, adult equiv.	3.942	3.968	4.13	3.699
(sd)	(1.90)	(1.88)	(2.38)	(1.98)
Farm operators, all households (share)	0.772	0.794	0.713	0.79
Farm operators, rural households (share)	0.919	0.943	0.888	0.882
Annual consumption per person, USD PPP, urban HHs	1600	2000	2246	1641
(sd)	(1912)	(2382)	(1856)	(1727)
Annual consumption per person, USD PPP, rural HHs	829.6	747.9	1008	674.9
(sd)	(1231)	(606)	(820)	(1040)

**Table 2:** Overview of productivity variable construction.

	Per person	Per hour
*Activity level productivity measures*		
Farming	Farm net revenue / (# own farm workers + predicted # hired in farm workers)	Farm net revenue / (hours worked by own farm and hired in workers)
Self employment	Firm profits / (# HH firm workers + # hired in firm workers)	*Not generated*
Wage employment	HH wage returns / # HH members participating in wage employment	HH wage returns / # hours worked for wages by HH members

*Sector level productivity measures*		
Agriculture	(HH net returns to farming + livestock + hired out ag wage labor) / (# hh members who participate primarily in ag sector)	(HH net returns to farming + hired out ag wage labor) / (hours worked on own farm + hours hired out for wages in ag)
Industry	(HH net returns to ind sector NFE + hired out ind sector wage labor) / (# hh members who participate primarily in ind sector)	(HH net returns to ind sector NFE + hired out ind sector wage labor) / (hours worked on own ind sector NFE + hours hired out for wages in ind sector)
Services	(HH net returns to ser sector NFE + hired out ser sector wage labor) / (# hh members who participate primarily in ser sector)	(HH net returns to ser sector NFE + hired out ser sector wage labor) / (hours worked on own ser sector NFE + hours hired out for wages in ser sector)

**Table 3:** Ratios between non-agriculture and agriculture in output per worker per year (productivity gaps), hours worked per year (employment gaps) and output per hour worked (per-hour productivity gaps).

	Per-person productivity gaps	Employment gaps	Per-hour productivity gaps
Ethiopia 2013–14	2.25	2.66	0.85
Malawi 2010–11	4.76	3.3	1.44
Uganda 2010–11	4.48	2.1	2.13
Tanzania 2010–11	4.2	2.22	1.9

**Table 4a:** Characteristics of own account and wage workers, rural adults.

	Ethiopia 2013–14		Malawi 2010–11		Tanzania 2010–11		Uganda 2010–11	
	Self	Wage	Self	Wage	Self	Wage	Self	Wage
*Agriculture* (share)	0.863	0.0588	0.886	0.326	0.817	0.0897	0.741	0.152
Hours/year, mean	553.3	631.5	423.1	432.2	561.5	382.6	638.3	722.9
Share female	0.516	0.39	0.527	0.439	0.53	0.379	0.568	0.491
Age, mean	40.03	39.66	39.17	37.21	41.15	39.3	40.65	39.8
Educ yrs, mean	1.51	1.614	4.899	4.589	5.502	5.13	5.416	4.633
Returns/year (positive), med	439.1	168.3	367.1	92.28	330.4	164	246.2	72.42
Returns/year (positive), mean	552.9	427.8	422.4	265.4	425	513.2	322.3	408.2

*Industry* (share)	0.0265	0.0079	0.298	0.0343	0.0506	0.0274	0.033	0.0643
Hours/year, mean	1129	1339	586.2	1371	490.5	1138	581.5	912.9
Share female	0.657	0.18	0.529	0.102	0.521	0.176	0.652	0.34
Age, mean	37.57	37.1	40.34	37.26	40.39	38.05	40.81	38.22
Educ yrs, mean	2.085	3.8	4.872	6.852	6.883	7.656	5.156	6.625
Returns/year (positive), med	260	1033	199.9	769	394.3	888.1	253.5	348.5
Returns/year (positive), mean	617.4	1782	423.9	1475	896.6	3189	690.9	2063

*Services* (share)	0.0834	0.0356	0.353	0.0643	0.256	0.0856	0.132	0.118
Hours/year, mean	1336	1480	926.7	1319	647.3	1433	1448	1240
Share female	0.559	0.253	0.399	0.207	0.526	0.269	0.418	0.359
Age, mean	37.51	35.97	36.36	38.86	39.23	38.9	38.88	39.64
Educ yrs, mean	2.601	8.975	6.484	9.811	6.77	9.214	7.397	9.455
Returns/year (positive), med	473.5	1946	291.6	1102	546.5	2049	633.7	937.7
Returns/year (positive), mean	1245	2785	674.7	1982	1737	4033	1562	2334

**Table 4b:** Characteristics of own account and wage workers, urban adults.

	Ethiopia 2013–14		Malawi 2010–11		Tanzania 2010–11		Uganda 2010–11	
	Self	Wage	Self	Wage	Self	Wage	Self	Wage
*Agriculture* (share)	0.0482	0.0154	0.284	0.204	0.257	0.0198	0.236	0.0632
Hours/year, mean	307.7	1641	183.5	860.6	354.9	1016	376.6	503.6
Share female	0.45	0.366	0.525	0.264	0.562	0.321	0.607	0.54
Age, mean	40.14	39.27	37.57	37.05	41.67	36.64	42.1	40.67
Educ yrs, mean	5.455	9.244	8.231	7.08	7.097	5.786	8.325	8.323
Returns/year (positive), med	336.3	2069	240.1	430.7	259.4	512.4	141.1	154.5
Returns/year (positive), mean	524.9	2833	303.8	1055	337.2	1468	279.9	457.7

*Industry* (share)	0.028	0.0931	0.104	0.0807	0.0842	0.0705	0.0462	0.0977
Hours/year, mean	1512	1880	1256	1814	557.5	1653	1530	1319
Share female	0.597	0.25	0.387	0.0787	0.424	0.15	0.451	0.235
Age, mean	39.78	36.49	36.81	37.1	38.24	36.88	40.75	37.13
Educ yrs, mean	6.627	8.575	9.387	10.73	7.638	8.942	9.149	9.797
Returns/year (positive), med	903.9	2367	1268	1964	755.1	2186	461.3	1432
Returns/year (positive), mean	3459	3717	2636	4559	1453	5820	1153	3914

*Services* (share)	0.195	0.316	0.516	0.26	0.397	0.245	0.238	0.242
Hours/year, mean	1692	1888	1232	1846	730.6	2084	2057	1845
Share female	0.494	0.417	0.472	0.286	0.583	0.306	0.576	0.425
Age, mean	37.59	36.02	34.71	36.73	38.61	37.32	40.54	38.26
Educ yrs, mean	6.951	10.92	9.349	11.36	8.158	9.765	9.261	11.91
Returns/year (positive), med	1549	2540	879.3	2076	1168	3074	1542	3331
Returns/year (positive), mean	3542	3990	3665	5891	2928	5665	2918	5814

**Table 5a:** Detailed sectors of non-farm self and wage employment, rural areas.

	Ethiopia 2013–14		Malawi 2010–11		Tanzania 2010–11		Uganda 2010–11	
	Self	Wage	Self	Wage	Self	Wage	Self	Wage
N (HHs or indivs) in sample								
Households	3776		2390		2583		2049	
Individuals		5941		3428		4331		3451
of which N participate	1263	1746	441	1354	1044	786	954	976
N firms (self) or jobs (wage)	1683	1956	469	1415	1404	856	1473	1102

Share of firms (self) or jobs (wage) by sub-sector								
Ag and primary prod share	0.0778	0.2240	0.0085	0.7760	0.0135	0.4050	0.0978	0.5340
Mining share	0.0297	0.0041	0.0107	0.0035	0.0135	0.0152	0.0129	0.0036
Manufacturing share	0.1600	0.0067	0.3820	0.0269	0.1410	0.0350	0.1320	0.0853
Electricity, utilities share		0.0041		0.0021		0.0117	0.0014	0.0018
Construction share	0.0119	0.0174	0.0085	0.0297	0.0071	0.0467	0.0020	0.0617
Commerce share	0.6350	0.0046	0.4990	0.0155	0.6620	0.1160	0.2630	0.0799
Transport, storage, comm. sh.		0.0072	0.0277	0.0064	0.0256	0.0397	0.0299	0.0299
Finance, real estate share	0.0018	0.0067		0.0021	0.0014	0.0187	0.0007	0.0000
Services share	0.0547	0.1190	0.0640	0.1330	0.1310	0.3080	0.1150	0.2030
Other industries share		0.0092		0.0000		0.0000		0.0000
Missing sector info share	0.0285	0.5970		0.0050	0.0043	0.0035	0.3460	0.0000

**Table 5b:** Detailed sectors of non-farm self and wage employment, urban areas.

	Ethiopia 2013–14		Malawi 2010–11		Tanzania 2010–11		Uganda 2010–11	
	Self	Wage	Self	Wage	Self	Wage	Self	Wage
N (HHs or indivs) in sample								
Households	1486		857		1263		584	
Individuals		2137		1457		2154		1085
of which N participate	526	929	340	657	730	597	362	379
N firms (self) or jobs (wage)	609	980	409	701	1011	629	583	426

Share of firms (self) or jobs (wage) by sub-sector								
Ag and primary prod share	0.0230	0.0418	0.0049	0.2870	0.0089	0.0461	0.0292	0.2460
Mining share	0.0066	0.0041	0.0024	0.0014	0.0237	0.0223	0.0120	0.0024
Manufacturing share	0.0755	0.0857	0.1490	0.0585	0.0940	0.0636	0.0823	0.0728
Electricity, utilities share		0.0224		0.0128	0.0040	0.0143	0.0069	0.0094
Construction share	0.0263	0.0776	0.0122	0.0542	0.0119	0.0715	0.0034	0.0892
Commerce share	0.7270	0.0612	0.6650	0.0485	0.5770	0.2050	0.3640	0.0657
Transport, storage, comm. sh.		0.0582	0.0562	0.0471	0.0307	0.1080	0.0377	0.0822
Finance, real estate share		0.0418	0.0122	0.0300	0.0079	0.0350	0.0034	0.0070
Services share	0.1150	0.4380	0.0978	0.4520	0.2340	0.4210	0.1610	0.4230
Other industries share		0.0316		0.0000	0.0020	0.0016		0.0000
Missing sector info share	0.0263	0.1380		0.0086	0.0059	0.0111	0.3000	0.0024

## Footnotes

1 Two other countries, Nigeria and Niger, are part of the LSMS-ISA data collection effort. I have not included Nigeria due to questionnaire incompatibilities with regards to self-employment labor supply. Niger is not included because its rural economy is heavily focused on livestock, and livestock labor inputs are not explicitly collected in the surveys.

2 These ratios were calculated using data from Gollin et al. (2014a) and World Bank classifications of countries by income.

3 For a description of how labor productivity growth can be decomposed into share-weighted labor productivity growth and productivity-weighted labor shifts across sectors, see McMillan et al. (2014).

4 Typically, input quality controls, including human capital, are used in productivity measurement.

5 Non-agricultural growth also contributes to agricultural growth (Irz and Roe, 2005). Empirically, agricultural growth has been shown to contribute more to poverty reduction than non-agricultural growth (Christiaensen et al., 2011).

6 The national account measure comes from the sector employment dataset published by the International Labor Organization (ILO) and sector value added measures from the United Nations (UN) National Accounts Statistics, accessed through the World Bank World Development Indicators database. Sector employment statistics are generated from population censuses or labor force surveys, with methodologies varying across countries. The Malawi agricultural labor share estimate is from Gollin et al. (2014b). The DHS measure of sector labor shares are based on the self-reported primary occupations of adult respondents who work and do not attend school. These are taken from McMillan and Harttgen (2014).

7 Following McMillan and Harttgen (2014), adulthood is assumed to begin at age 25 to avoid confounding labor shares and educational attainment. Labor shares are robust to the adulthood threshold used.

8 In most LSMS-ISA surveys, respondents are not asked to name their primary occupations explicitly.

9 Ethiopia is omitted from this analysis because the questionnaire administration is highly concentrated in a two-month period, with very low coverage in other months.

10 Results can be shared upon request.

11 In the Malawi survey, industry-sector self-employment firms tend to be involved in rudimentary value addition activities like beer brewing or brick making or basket weaving. In Malawi, basic roadside selling of food products was coded as manufacturing, whereas in other countries, these activities are usually coded to the service sector.

12 This impression is based on my own experience shadowing LSMS-ISA survey teams in Ethiopia during the 2012–2013 round of surveys, and on discussions with survey teams Ethiopia. “Ganyu” wage labor from Malawi and “Productive Safety Net Program” labor from Ethiopia were categorized as agricultural based on consultations with survey teams.
